# Oligomerization of Selective Autophagy Receptors for the Targeting and Degradation of Protein Aggregates

**DOI:** 10.3390/cells10081989

**Published:** 2021-08-05

**Authors:** Wenjun Chen, Tianyun Shen, Lijun Wang, Kefeng Lu

**Affiliations:** 1 Department of Neurosurgery, State Key Laboratory of Biotherapy, West China Hospital, Sichuan University, Chengdu 610041, China; chenwenjun@stu.scu.edu.cn (W.C.); shentianyun123@outlook.com (T.S.); WangLi-jun@hotmail.com (L.W.); 2 Department of Neurology, Shanxi Provincial People’s Hospital, Taiyuan 030012, China

**Keywords:** receptors, autophagy, proteasome, ubiquitin, p62, Dsk2, Cue5, TAX1BP1

## Abstract

The selective targeting and disposal of solid protein aggregates are essential for cells to maintain protein homoeostasis. Autophagy receptors including p62, NBR1, Cue5/TOLLIP (CUET), and Tax1-binding protein 1 (TAX1BP1) proteins function in selective autophagy by targeting ubiquitinated aggregates through ubiquitin-binding domains. Here, we summarize previous beliefs and recent findings on selective receptors in aggregate autophagy. Since there are many reviews on selective autophagy receptors, we focus on their oligomerization, which enables receptors to function as pathway determinants and promotes phase separation.

## 1. Introduction

Protein misfolding and the subsequent aggregation caused by various stressors can be cytotoxic to cells and may lead to cell death. To maintain cellular homeostasis, organisms use various protein quality-control pathways to either repair misfolded proteins or target them for degradation if misfolding or aggregation persists [1,2]. In general, protein substrates with different statuses, soluble or aggregated, are targeted and degraded by proteasome and autophagy pathways, respectively [3]. Soluble substrates are mainly degraded through the proteasome pathway; tightly folded protein aggregates, however, are unable to traverse the narrow opening of the proteasome and are thus resistant to proteasomal degradation and may accumulate in cells as ubiquitinated species [4,5]. For clearance, aggregation-prone substrates are typically linked to the autophagosome and then degraded in lysosomes through the selective autophagy pathway. Although targeted differently, soluble and aggregated substrates are both modified with polyubiquitin chains, which were initially thought to form only on proteasomal substrates [6,7,8]. This common modification of different substrates generates a puzzle regarding how this degradation decision is made, especially considering that both pathways utilize ubiquitin-binding proteins as substrate receptors. For example, yeast proteasome receptors Dsk2 and Rad23 bind to soluble polyubiquitinated proteins while autophagy receptors such as p62 or TOLLIP (in humans) and Cue5 (in yeast) recognize ubiquitinated aggregates [9,10,11,12,13,14,15,16].

In yeast, Dsk2 plays a key role as a receptor in the ubiquitin-proteasome system (UPS) by binding to soluble substrates via a ubiquitin-binding UBA domain and targeting them to proteasome via a ubiquitin-like (UBL) domain. In contrast, autophagy receptor Cue5 harbors a different ubiquitin-binding domain, a CUE domain, and a binding motif for Atg8 (Atg8-interacting motif, AIM; in humans, this is called the LC3-binding motif, LIR), a ubiquitin-like protein conjugated to autophagosomal membranes. Dsk2 and Cue5 transport soluble and insoluble substrates to the proteasome and autophagosome respectively through UBL or AIM modules, which target cargo-bound receptors to the proteasome or autophagosome. However, substrates for p62 are not fundamentally different from those of the UPS in mammalian cells. P62 binds to ubiquitinated proteins by its UBA domain and delivers substrates to the lysosomes for degradation. In addition, p62 can transport ubiquitinated proteins to the proteasome for degradation [17], which raises the question of how receptors recognize substrates with different statuses. Several theories were proposed to explain the selectivity of ubiquitinated substrates by diverse receptors. It was suggested that soluble and aggregated proteins are modified with different ubiquitin chain types; for example, soluble substrates were speculated to be conjugated with Lys48-linked polyubiquitin chains and aggregates tagged with Lys63-linked chains [18,19,20,21,22]. However, autophagy receptors show no obvious preference for binding to Lys63-linked polyubiquitin chains over Lys48-linked chains. OPTN with Lys48-linked poly-Ub chains is predominantly targeted for autophagy-dependent degradation [23]. Thus, the real determinants of the two degradation pathways need to be clarified [9,10,11,12,13,14,15].

Autophagy is a species-conserved pathway for the degradation of cytoplasmic materials in lysosomes (called vacuoles in yeast) and the recycling of nutrients. Autophagy was first characterized as a bulk degradation pathway under starvation conditions [24,25]. However, recent findings made it clear that autophagy also selectively targets intracellular cargoes such as protein aggregates, damaged mitochondria, excess peroxisomes and ribosomes and invading pathogens [6,26]. Many studies show that selective autophagy plays an important role in cellular homeostasis, and abnormalities in autophagy can cause neurodegenerative diseases, cancers and immune dysfunction. Here, we focus on the selective autophagy degradation of protein aggregates mediated by receptors. Furthermore, we describe key features for these receptors to function in discriminating cargo aggregates from soluble proteins.

## 2. A Brief History of Selective Autophagy

In 1973, Bolender and Weibel discovered that the smooth endoplasmic reticulum could be specifically degraded by autophagy [27]. Ten years later, in 1983, Veenhuis and Dunn first reported selective peroxisomal autophagy (pexophagy) in Hansenula [28]. In 1985, Dice et al. found that lysosomes can selectively degrade soluble proteins and ribonucleases, namely, chaperone-mediated autophagy (CMA) [29]. Later, in 1987, Mortimore et al. reported that autophagy selectively degraded ribosomes in hepatocytes [30]. In 2004, mitophagy was proposed by Lemasters et al. [31]. Yoshimori and colleagues found that Streptococcus A in the cytoplasm is immediately captured by autophagic vesicles and eventually fuses with lysosomes for destruction [32]. Meanwhile, Deretic demonstrated that autophagy is involved in the intrinsic immunity of host cells to a variety of pathogens [33]. Currently, there is considerable evidence to support the notion that the process is also highly specific.

In selective autophagy, an important role is played by autophagy receptors such as p62 and NBR1. The discovery of selective autophagy receptors was a breakthrough in the autophagy field, providing the necessary mechanistic underpinning of the formation of an autophagosome selectively around the cytosolic cargo. ATG11 (cvt9) was the first protein found to be essential for selective autophagy. It is responsible for the vesicular transport of α-aminopeptidase and peroxisomes through the Cvt pathway. In 2010, Ohsumi’s group identified the second Cvt cargo receptor, Atg34, which is required for the efficient trafficking of Ams1 to vacuoles [34]. However, it is the protein aggregation pathway that really sheds light on the selective autophagy machinery in mammalian cells [35]. In 1996, Vadlamudi discovered that the p62 protein binds to ubiquitinated proteins [36]. Approximately 10 years later, Johansen found that p62 binds to LC3, assembles into autophagosomes, and eventually degrades along with ubiquitinated proteins in autophagic lysosomes [10]. The recognition of the first autophagy receptors in yeasts and mammals greatly facilitated the search for autophagic receptors. In 2009, several groups working together identified NBR1 as a ubiquitin- and LC3/GABARAP-binding protein, and a major aggrephagy factor [11]. The realization that the mammalian genome encodes a number of proteins interacting with both LC3/GABARAP and the cargo further strengthened the hypothesis of selective autophagy machinery.

## 3. Autophagosome Formation and Atg8/LC3 Scaffolds as Platforms for Receptor Cargo Recruitment

**Figure 1:**
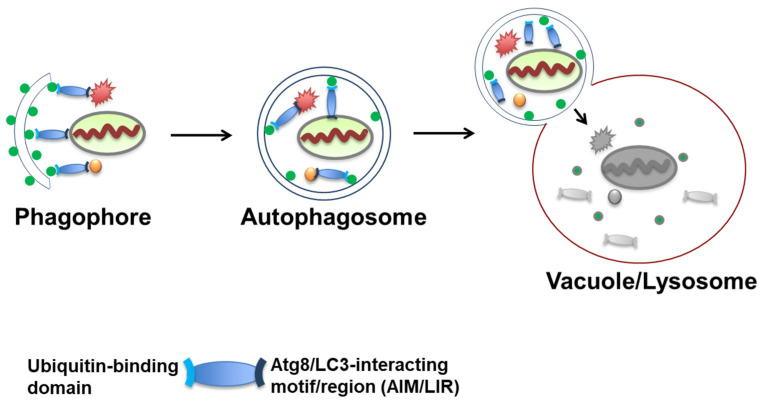
Autophagy pathway begins with the formation of a double-layered isolation membrane (phagophore), which sequesters autophagy cargo such as protein aggregates or organelles. Selective autophagy receptors targets cargos to Atg8/LC3-conjugated isolation membranes. After expansion, the fully formed autophagosome fuses into vacuoles or lysosomes to degrade and recycle their cargoes.

Autophagy is a very evolutionarily conserved pathway for the lysosomal degradation of long-lived proteins, large aggregates, and whole organelles (Figure 1). Autophagy in yeast is usually maintained at a very low background level under rich medium culture conditions and it is dramatically induced under starvation or rapamycin treatment. In mammalian cells, changes in autophagy levels can be cell type- and context-specific, although in most cases, autophagy is constitutively activated [37,38,39,40,41].

The autophagy process begins with the formation of a double-membrane vesicle called an autophagosome (0.5–1.5 μm), to which Atg8 (LC3 in mammalian cells) is conjugated to engulf cytosolic cargoes. In yeast, autophagosomes grow from a single spot called the phagophore assembly site (PAS), also known as preautophagosomal structure, where the steps of initiation, nucleation, elongation and closure inside a cup-shaped double-layer membrane to produce the phagophore successively occur. Yeast genetic screens led to the identification of 40 autophagy-related (ATG) genes that have various roles in the different steps of autophagosome formation [42,43,44]. The initiation of autophagy is regulated by the Atg1/Atg13/Atg17 kinase complex, which is inhibited by the target of rapamycin (TOR) kinase under rich medium culture conditions. In the next step, phagophore nucleation at the PAS is controlled by a lipid kinase complex containing Vps34 and regulatory subunits Atg14, Atg6/Vps30, and Vps15. The subsequent elongation step is controlled by Atg9, the only transmembrane protein in the autophagy pathway that provides a lipid membrane for the expanding phagophore by shuttling between various vesicle compartments and PAS depending on Atg1 and Vps34. Recently, Atg9 was found as a lipid scramblase to promote the translocation of phospholipids from the cytoplasmic to the luminal leaflet of liposomes/autophagosomes, thus driving the enlargement of autophagosome membranes [45]. In addition to Atg9, two highly conserved ubiquitin-like protein (Atg12 and Atg8) conjugation systems, Atg12-Atg5 and Atg8-phosphatidylethanolamine (PE), also contribute to expansion and closure of phagophore/isolation membrane [46,47,48,49,50]. Atg8-PE in the outer leaflet of the vesicle lipid bilayer leads to the hemifusion of the two separate membranes, which is realized by tethering together the outer leaflets of two lipid vesicles [46,51]. Eventually, Atg8-PE promotes autophagosome membrane expansion. Furthermore, the Atg8-PE has also been found to be important for the closure of isolation membranes to form sealed autophagosomes, indicating that Atg8-PE may possess membrane fission function in addition to its role in membrane fusion [46,51]. However, studies also showed that autophagosomes can correctly seal in the absence of Atg8-PE, which does not support the role of Atg8-PE in the closure of autophagosomal membranes [52,53,54]. Recently, several studies have shown that the endosomal sorting complexes required for transport (ESCRT) machinery plays a pivotal role in the closure of autophagosomal membranes [55,56,57,58]. Using the HaloTag-LC3 autophagosome completion assays, Takahashi et al. found that ESCRT-III component CHMP2A traffics to the phagophore and promotes the separation of the inner and outer autophagosomal membranes, which eventually leads to the formation of sealed autophagosomes [59]. Furthermore, Zhen et al. found that ESCRT-III machinery-mediated phagophore closure is pivotal for selective mitophagy [60].

Ubiquitin (Ub) is a small protein containing 76 amino acids and is highly conserved from yeast to humans. It is tightly folded to form a globular structure comprising a five-stranded beta-sheet wrapped around a central helix. Ubiquitin is first synthesized as a precursor protein. Subsequent proteolytic cleavage exposes its active C-terminal glycine, which allows for ubiquitin to be conjugated to a lysine (or N-terminal methionine) in the substrate protein or in the first ubiquitin moiety. A cascade of catalytic enzymes involving activating (E1), conjugating (E2) and ligating (E3) enzymes generate ubiquitin conjugates containing either multiple mono-Ub or poly-Ub chains (mostly Lys48, Lys63, or linear) [61,62,63]. Diverse types of Ub modifications confer diverse functions, such as the regulation of cytoplasmic membrane receptor endocytosis, targeting the proteins for degradation or a role in signaling complex assembly [64,65,66,67,68]. Proteins containing Ub-binding domains (UBDs) could act as ubiquitin receptors through noncovalent interactions with Ub. Ub conjugates can be reversed by a large class of deubiquitinating proteases (DUBs) that cleave ubiquitin moieties from their substrate. Atg12 was the first ubiquitin-like protein (UBL) to be identified in the autophagy pathway [69]. In contrast to Ub, it is synthesized as a C-terminal glycine-exposed form. Autophagy core protein Atg7 (E1) directly transfers Atg12 onto Atg10 (E2) in a cascade manner. Atg12 is lastly conjugated to Atg5. The Atg12-Atg5 conjugate then recruits factors important for phagophore elongation and closure. The Atg12-Atg5 conjugate works in conjunction with dimerized coiled-coil protein Atg16, which promotes the association of this conjugate with PAS and phagophore elongation. The main role of the Atg12-Atg5-Atg16 complex is to function as E3 for Atg8-PE conjugation. Atg8, another UBL that functions in autophagy, similar to Ub, is synthesized as a precursor and is cleaved by cysteine protease Atg4. The mature form of Atg8 with an exposed C-terminal glycine is activated by and conjugated to Atg7 (E1), transferred to Atg3 (E2) and lastly conjugated to PE lipids incorporated into the phagophore with the help of an Atg12-Atg5 conjugate. Conjugation of Atg8 to phagophores is essential for its expansion and is important for its function as a platform for the recruitment of its cargo, which is mediated by autophagy receptors [70,71,72,73].

## 4. Autophagy Receptors for the Degradation of Protein Aggregates

**Figure 2:**
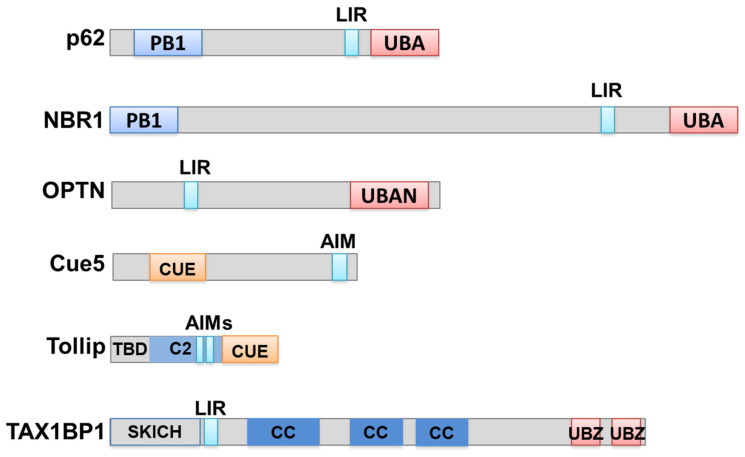
Scheme of functional domains of autophagy receptors in aggrephagy. Essential domains are featured on the receptor proteins. Features: PB1, Phox and Bem1 domain; LIR, LC3-interacting region; UBA, ubiquitin-associated domain; UBAN, ubiquitin-binding domain in ABIN proteins and NEMO; CUE, coupling of ubiquitin conjugation to ER degradation domain; AIM, Atg8-interacting motif; TBD, Tom1-binding domain; C2, C2 domain; SKICH, SKIP carboxyl homology domain; UBZ, ubiquitin-binding zinc finger.

On the basis of the nature of the cargoes, several types of selective autophagy were found: aggrephagy (clearance of protein aggregates), mitophagy (clearance of damaged mitochondria), ribophagy (clearance of excessive ribosomes), xenophagy (clearance of invading pathogens), pexophagy (clearance of peroxisomes), reticulophagy (clearance of the endoplasmic reticulum), nucleophagy (clearance of the nuclear envelope), and lipophagy (clearance of the liposomes) [9,10,11,12,13,14,15]. Autophagy receptors play critical roles in the recognition, recruitment and selective autophagic substrate transport of the substrates. However, different autophagy receptors contain different functional domains that determine their different functions. Here, we mainly focused on autophagy receptor function during aggrephagy (Figure 2).

As a signal for destruction, Ub tags both soluble substrates for the proteasome and insoluble aggregates for autophagy degradation. Known aggrephagy receptors include p62, NBR1 (neighbor of BRCA1), OPTN (optineurin), the CUET family (Cue5 in yeast and Tollip in humans) and Tax1-binding protein 1 (TAX1BP1) [11,12,74,75,76,77,78,79,80,81,82]. These receptors all harbor both Ub-binding domains for cargo targeting and Atg8 interacting motif/LC3-interacting regions (AIMs or LIRs, with the conserved sequence W/F/YxxL/I/V) for autophagosome recruitment.

p62, previously known as an adaptor protein for PKC, MEK5 and NF-κB signaling [83,84], is the first and best-characterized autophagy receptor for the clearance of aggregates. p62 has a classical modular structure as an autophagy receptor: a C-terminal Ub-binding UBA domain, an LIR for LC3 binding and an N-terminal PB1 domain that could confer association with itself or other proteins. The interaction of p62 with the Ub moiety is essential for its receptor function. In autophagy-deficient cells, p62 accumulates in Ub-positive aggregate sites and p62 is a protein marker for cellular inclusion bodies within cells from patients with various forms of neurodegenerative diseases [85]. The UBA domain of p62 binds to several kinds of ubiquitin conjugates with little preference for Lys63-polyUb chains. The affinity of UBA for Ub is regulated by UBA phosphorylation [86].

NBR1 was first identified as a signal transduction adaptor. Its domain arrangements are very similar to those of p62: both have an N-terminal PB1 (Phox and Bem1p) domain, middle LIRs and a C-terminal UBA domain [11] (Figure 2). However, NBR1 is much larger than p62. Similar to p62, NBR1 binds to ubiquitin and LC3 and mediates the autophagic degradation of ubiquitinated substrates. Genetic analysis showed that NBR1 is an ancestral protein with homologs throughout all kinds of eukaryotic species; in contrast, p62 is mostly confined to metazoans. In knockdown and overexpression experiments, NBR1 was a major autophagy receptor for aggrephagy and pexophagy [11].

Optineurin is a cytosolic protein encoded by the OPTN gene, and OPTN functions in signal transduction, membrane-vesicle trafficking, cell division and cytokine secretion [87,88,89]. OPTN mutations are linked to glaucoma and neurodegenerative amyotrophic lateral sclerosis (ALS), and are frequently detected in pathological protein inclusions. The OPTN protein possesses LIR, C-terminal UBAN, and UBZ domains, and several coil–coil domains mediating its oligomerization. With its C-terminal UBAN and UBZ domains, OPTN binds to misfolded proteins and aggregates in both Ub-dependent and -independent manners for autophagy clearance. In addition to its function in aggrephagy, OPTN could also promote the autophagy clearance of Salmonella, which is regulated by phosphorylation catalyzed by TBK1 [77,88].

The CUET family includes yeast Cue5 and human Tollip, which are conserved from yeast to humans. Although p62 functions as an autophagic receptor in aggrephagy in humans, its homolog in yeast has not been found. Considering the conservation of the autophagy pathway from yeast to humans, a conserved aggrephagy receptor family from yeast to humans probably exists. Indeed, Cue5 was the first aggrephagy receptor in yeast, harboring a ubiquitin-binding CUE domain. Human CUE domain protein Tollip is functionally similar to yeast Cue5, indicating that the CUET family proteins represent a new and conserved class of autophagy receptors. Both Cue5 in yeast and Tollip in human cells mediate the efficient clearance of aggregation-prone polyQ proteins derived from human HTT/huntingtin [12]. Tollip is potentially more potent in polyQ clearance than p62 in HeLa cells is, suggesting that Tollip, also implicated in innate immunity, may be significant for human health and disease through its function as an aggrephagy receptor [80].

TAX1BP1 is an autophagy receptor protein that clears aggregates [82] and mitochondria [90], and was first characterized in the context of ubiquitin-driven xenophagy [91]. In addition, TAX1BP1 is highly conserved, and plays many essential functions in human cells, including the negative regulation of the inflammatory and antimicrobial responses mediated by NF-κB and IRF3 signaling, the inhibition of apoptosis, and transcriptional coactivation [92]. TAX1BP1 is highly and specifically expressed in the brain, and TAX1BP1 mediates clearance of a broad range of cytotoxic proteins, indicating its therapeutic potential in neurodegenerative diseases [82]. TAX1BP1 is a member of the CALCOCO gene family and contains a SKICH domain, a canonical LC3-interacting region (LIR), and coiled-coil domains while differing in its primary sequence and in the conservation of its C-terminal zinc finger (ZF) domains across various species [91]. The N-terminal SKICH domain mediates constitutive membrane association with a poorly defined function [93,94]. The two zinc fingers, localized in its C-terminus, function as novel ubiquitin-binding domains (UBZ, ubiquitin-binding zinc finger) that can bind to ubiquitin and TRAF6 with Lys63-linked chains [95,96]. The central part is three coiled-coil regions and is predicted to mediate oligomerization [97].

In brief, the proposed role of these receptors in aggrephagy is to bridge LC3/GABARAP family members with ubiquitinated substrates (Figure 1).

## 5. Pivotal Role of Oligomerization in Autophagy Receptor Function

Since the discovery of the ubiquitin–proteasome system, it has been well established that proteasomal degradation is mainly responsible for eliminating abnormal cellular proteins, especially misfolded proteins, under stress conditions such as heat shock. Misfolded proteins are recognized and bound by ubiquitin ligases and then modified by polyubiquitin chains, which become targeting signals for ubiquitin receptors to escort substrates to the proteasome for degradation. However, the discovery of autophagy receptors suggests that the autophagy pathway both functions under starvation conditions and eliminates protein aggregates modified with ubiquitin. Currently, it is clear that the proteasome and autophagy are both protein quality-control pathways that enable cells to maintain homeostasis [12,81]. As both pathways rely on the recognition of ubiquitin moieties attached to substrates by their respective receptors, how the pathway choice is made is a puzzling question.

After the discovery of different types of ubiquitin modifications, including monoubiquitin and polyubiquitin chains with different topologies, it was proposed that different types of ubiquitin modifications determine the different fates of the conjugates, i.e., proteasome or autophagy degradation. For example, some studies showed that ubiquitin-binding autophagy receptors prefer Lys63-linked polyubiquitin chains over Lys48-linked chains [9,10,11,12,13,14,15]. A prevailing view in the protein quality control field indicates that proteasome degradation and autophagy-dependent degradation pathways are independent, and that the degradation choice is made at the ubiquitin modification level. However, all types of Ub chains in autophagy-deficient cells accumulate, suggesting that autophagy has little selectivity for substrates with different types of ubiquitin chains. This is further supported by the observation that the autophagy pathway is still fully operational for the clearance of insoluble ubiquitinated substrates in cells deficient in the formation of different polyubiquitin chains [81]. Second, known autophagy receptors, such as p62 and NBR1, show little preference for binding to Lys63-linked ubiquitin chains, even though some studies showed no differences in their binding to different types of ubiquitin chains. The recently identified CUET family (Cue5 in yeast and Tollip in humans), a conserved receptor family for selective autophagy, has no selectivity for ubiquitin chain types. Third, misfolded proteins eliminated by the proteasome and by selective autophagy are modified by the same ubiquitination enzymes. Furthermore, the finding that Tollip functions as an autophagy receptor in addition to its roles in ubiquitin-dependent endocytosis and innate immunity signaling exemplifies the complexity of protein destruction networks and indicates that the fate of a protein is not solely determined at the ubiquitin-modification level [12,98,99]. Combining all of these previous studies, the hypothesis that the degradation choice for proteasome and autophagy pathways is made at the level of ubiquitin-binding adaptors comes to the forefront.

Therefore, the question is: what is the decision-maker? Proteolytic pathways, the proteasome and autophagy, coexist in yeast. *Saccharomyces cerevisiae* was used as a genetically tractable model organism that was combined with biochemical assays and synthetic biological approaches to uncover the decision-maker for the degradation-pathway choice. The answer was clarified by showing that proteasome-ubiquitin receptor Dsk2 and autophagy-ubiquitin receptor Cue5 have different properties. Both pathway receptors bind to polyubiquitin chains without discriminating between chain topologies. Proteasome pathway receptor Dsk2 has a higher affinity for ubiquitin moieties than autophagy receptor Cue5 does. In vivo evidence showed that Cue5 readily binds ubiquitinated substrates when Dsk2 is absent, but it is completely blocked from binding in the presence of Dsk2 overexpression. Further direct in vitro competition experiments showed that Dsk2 could readily stop Cue5 from ubiquitin binding; however, Cue5 could only weakly hinder Dsk2 from ubiquitin binding. Proteasome pathway receptor Dsk2 acts as a solitary protein, whereas autophagy receptor Cue5 forms oligomers and is present at higher levels [81].

To unequivocally define the essential features of a functional autophagy receptor and to reject previous models, synthetic receptors were designed. Interestingly, Cue5 oligomerization is mediated by its CUE domain, a characteristic property found in a class of CUE domain-containing proteins, such as Vps9, in endocytosis. For Vps9, CUE domain-mediated dimerization promotes ubiquitin interactions [100]. Similarly, residues in Cue5′s CUE domain are required for ubiquitin-binding and its self-interaction (oligomerization). Cue5 but not Dsk2 can assemble into higher-ordered oligomers in other autophagy-ubiquitin receptors, such as p62 and NBR1.

To unravel whether oligomerization is a general necessity for autophagy-receptor functionality, a whole set of artificially constructed receptors using a synthetic biology approach were tested for their capability as autophagy receptors [81]. Most remarkably, a synthetic receptor based on GFP harboring an 8-amino acid domain that binds to autophagy protein Atg8, further amended with a ubiquitin-binding domain and an artificial oligomerization domain, was found to function like natural autophagy receptor Cue5 in yeast [81]. However, its monomeric equivalent could specifically mediate the clearance of aggregated ubiquitin conjugates.

**Figure 3:**
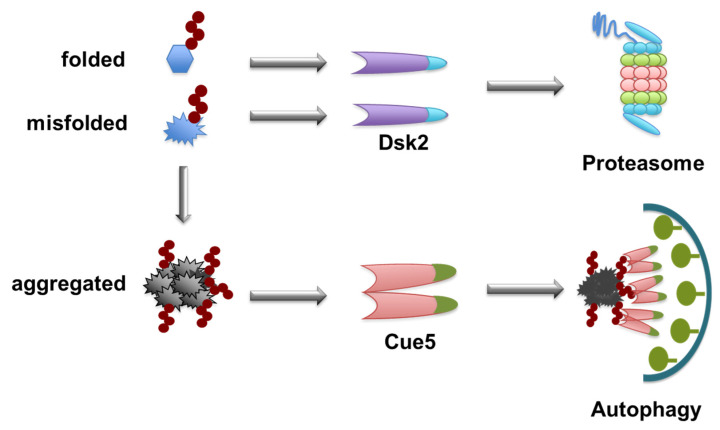
Proteasome receptor Dsk2 and autophagy receptor Cue5 function in the clearance of soluble and aggregated substrates. Monomeric Dsk2 binds soluble or slightly misfolded substrates modified with ubiquitin chains for proteasome degradation. When overwhelming numbers of misfolded proteins accumulate into stable aggregates, oligomeric Cue5 then binds to and targets substrates for autophagy degradation.

The different ubiquitin-binding affinities and oligomerization features of receptors make substrate selection spontaneous, and later events depend on the status of the substrate. This is an economical method for cells to discriminate different substrates without involving additional ubiquitination enzymes for different substrates. The shared upstream enzymes also indicate that the ubiquitination of aggregates probably begins when the protein is still soluble or slightly misfolded. It is not clear whether more ubiquitin modifications continue after protein aggregates are formed. How does oligomerized Cue5 gain the upper hand in binding to ubiquitinated aggregates? This may be exemplified by the way in which p62 selectively binds to autophagosome membrane-conjugated LC3. Oligomerized p62 only binds to LC3 attached to the autophagosome membrane with the highest affinity; in cases of mutations causing monomer p62 or free LC3, this binding is dramatically reduced. It is assumed that once the receptor oligomerizes, its binding to the autophagosome-clustered LC3 is highly stabilized, which enables p62 to select it for the autophagosome membrane. This kind of stabilized binding is especially beneficial for autophagy receptors with oligomerization to select their aggregated substrates. Although ubiquitin modification is probably not increased much when aggregates form, its clustering on the surface of aggregates sufficiently stabilizes the binding between substrates and receptors to target autophagosomes. This highlights its zipper-like property of binding to accreted aggregates, bundled receptors and membrane-conjugated LC3 (Figure 3). The oligomerization of receptors can be stress-induced. For example, oxidation of p62 at cysteine sites is triggered by oxidative stress, which allows for its oligomerization and enhances the targeting and autophagic degradation of p62-bound ubiquitinated substrates [101]. Mutations of p62 such as ALS-related K102E can impair its oligomerization, indicating the physiological importance of p62 oligomerization [101]. Furthermore, NBR1 homologs are found throughout the eukaryotes, while p62 is confined to the metazoans, and Arabidopsis thaliana NBR1 (AtNBR1), similar to p62, was found to homo-polymerize by the PB1 domain [102].

## 6. Role of Oligomerization in Autophagy Substrates

In addition to conferring the capability of autophagy receptors, oligomerization features are also important for defining substrates for autophagy degradation. The cytoplasm-to-vacuole targeting (Cvt) pathway is a type of selective autophagy found specifically in yeast that constitutively and selectively transports hydrolases, such as Ape1, Ams1, and Ape4 to vacuoles through autophagy [103,104,105,106]. Among cargos in the Cvt pathway, Ape1 is the only cargo to function as a template for Cvt vesicle recruitment and formation. Many biochemical and cell biological studies elucidated the process of Ape1 transportation to vacuoles through the Cvt pathway. After synthesis, a precursor form of Ape1 called prApe1 quickly folds as a homododecamer. The propeptide of prApe1 further assembles the dodecamers into a larger aggregate named the Ape1 complex, which acts as a platform for Cvt vesicles. The Ape1 complex is then bound by specific autophagy receptor Atg19 via an interaction between the propeptide and the coiled-coiled (CC) domain of Atg19. Receptor Atg19 then recruits Atg8 through the AIM located in the C-terminus of Atg19, followed by recruitment of the autophagy machinery and autophagosome-membrane formation, which is eventually fused with the vacuole and released inside the Ape1 complex. In vacuoles, prApe1 is digested into a mature form (mApe1) through cleavage of the propeptide. Since prApe1, but not mApe1, is a selective cargo in the Cvt pathway, oligomerized prApe1 is thought to be the determinate for discrimination. Indeed, the mechanism of this selection is explained by the fact that sequestration of prApe1 into oligomers supports the efficient binding to receptor Atg19 and its delivery to vacuoles. Artificially designed prApe1 lacking its N-terminal propeptide, which is transformed into a monomer instead of an oligomer, could not be efficiently targeted by Atg19 and then failed to be transported to the vacuole. On the other hand, fusion of the prApe1 N-terminal propeptide to the originally monomeric cytosolic proteins forces them into a large complex and promotes their vacuole delivery by autophagy. This clearly demonstrates the importance of oligomer formation of autophagy substrates.

Another example shows the importance of oligomerization in determining autophagy substrates, namely, LC3-binding proteinTBC1D25/OATL1 [103]. TBC1D25 was originally described as an inhibitor protein involved in the maturation step of autophagosomes by binding to LC3. However, it is not a substrate of p62, although both proteins bind to LC3 on the phagophore membrane. Researchers discovered the molecular basis of TBC1D25 escape from being an autophagy substrate by the chimeric analysis and artificial manipulation of TBC1D25 and p62. Results showed that chimeric TBC1D25, which maintains its interaction with LC3, becomes an autophagy substrate only when it is forcibly oligomerized by the addition of the PB1 domain from p62. Altogether, these results indicate the key role of oligomerization in determining substrates for autophagy degradation.

## 7. Segregation of Aggrephagy Cargos

As described above, both the oligomerization of autophagy substrates and the oligomerization of autophagy receptors are important for aggrephagic cargo formation and autophagic degradation. For example, p62 bodies are round protein bodies formed in cultured cells, accompanying ubiquitin-positive protein aggregates [74]. The genetic ablation of p62 blocks the appearance of ubiquitin-positive protein aggregates in hepatocytes and neurons, indicating that p62 plays an important role in inclusion body formation [75]. The polymerization of p62 via the PB1 domain is necessary for the formation of protein aggregates and their degradation by autophagy, which also supports this view [74]. P62 alone cannot form p62 bodies. The mechanisms involved in the formation of aggrephagy cargos had not been very clear until the concept of the liquid-liquid phase separation of biological macromolecules was proposed [107]. It is increasingly clear that many subcellular structures and compartments are results of phase-separation reactions [108]. In 2012, Li et al. reconstituted a liquid-liquid phase-separation phenomenon in vitro and established a natural phase-separation system in vivo [109], which suggests that the phase separation of biomolecules driven by multivalent interactions may be prevalent in cells. Multivalent interactions can be mediated by oligomerization domains such as the PB1 domain in p62 [110], by intrinsically disordered proteins (IDPs) [108], and alternatively by protein modifications including polyubiquitination, polySUMOylation or poly(ADP-ribosyl)ation at one site [108,109,111,112]. Taken together, these results suggest that all of these proteins, including misfolded proteins with polyubiquitination and multivalent UBA domains caused by p62 oligomerization, can create multivalent domains. Thus, p62 body formation relies on the oligomerization of the p62 protein and its binding to ubiquitinated proteins. Individual molecules can be highly mobile within p62 bodies, which undergo internal rearrangement and exchange molecules with the surrounding cytosol. Thus, aggrephagy cargos are segregated by liquid–liquid phase separation. Recently, it was shown that cytosolic p62-containing droplets formed by liquid–liquid phase separation are sequestered by autophagosomes and degraded by autophagy [113]. The surface of droplets serves as liquid assembly platforms for double-membrane autophagosomes through partial wetting, as the tension of droplet surface supports the formation of autophagosomal membrane sheets [113]. It highlights the importance of wetting in cytosolic compartmentalization by liquid–liquid phase separation involving receptors like p62 and in droplet removal by autophagy.

Another autophagy receptor, NBR1, may share the same mechanism since they have very similar characteristics. In addition, these two autophagy receptors can cooperate with each other in the selective autophagy of ubiquitinated targets. NBR1 can interact with p62 through the PB1 domain in each protein, suggesting that these two receptors may function together [114]. The PB1 domain of p62 or NBR1 plays a key role in the execution and regulation of selective autophagy, while other autophagy receptors do not have such structures [11,115]. The hypothesized mechanism is that other autophagy receptors may acquire this ability through cooperation with p62 or NBR1. For example, OPTN can be ubiquitinated by HACE1, which can specifically interact with the UBA domain of p62. The ubiquitinated OPTN-p62 complex can activate cellular autophagy [23]. Future investigations are necessary to test this interesting possibility.

## 8. Monomer and Oligomer Status of the Same Receptors Confers Dual Functions in Two Degradation Pathways

**Figure 4:**
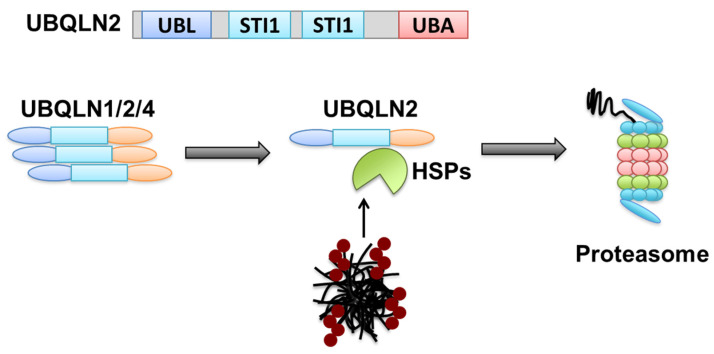
UBQLN2 functions in both proteasome and autophagy degradation pathways depending on its status. When UBQLN is held in homo or heterooligomers, it could target and mediate autophagy substrates. Under stress conditions, UBQLN2 is released and acts as a proteasome receptor for misfolded proteins disassembled by HSP70/HSP110 chaperones.

It is interesting that the two degradation pathways, proteasome and autophagy do not always use their respective receptors to target their substrates. This is exemplified by UBQLN2 (ubiquilin-2), which was first found in amyotrophic lateral sclerosis (ALS) [116,117]. The ubiquilin family, with 4 members, is the human homolog of yeast proteasome receptor Dsk2 [118]. It contains an N-terminal UBL domain for interaction with the proteasome and a C-terminal UBA domain for binding to polyubiquitinated proteins. Although previous studies discovered several disease-linked mutations in UBQLN2 involved in aggregate formation and related neurodegenerative diseases, its role and mechanism in the clearance of aggregates are unknown. Recently, it was shown that UBQLN2 functions together with the HSP70-HSP110 disaggregate machinery to clear protein aggregates from cells through the proteasome pathway. The aggregates are first disassembled by HSP70 complex chaperones before being recognized by UBQLN2 and linked to the proteasome for degradation. The function of UBQLN2 is regulated by its homologs UBQLN1 and 4, which can bind UBQLN2 through middle STI1 domains. When UBQLN2 is bundled with UBQLN1, UBQLN4 or itself, its function in the proteasome is inactive while it gains the capability to work as an autophagy receptor. Under stress conditions, HSP70/HSP110 chaperones disassemble and pull aggregated proteins apart, which allows for the released monomeric UBQLN2 to bind to and shuttle substrates to the proteasome for degradation (Figure 4).

## 9. Conclusions

**Figure 5:**
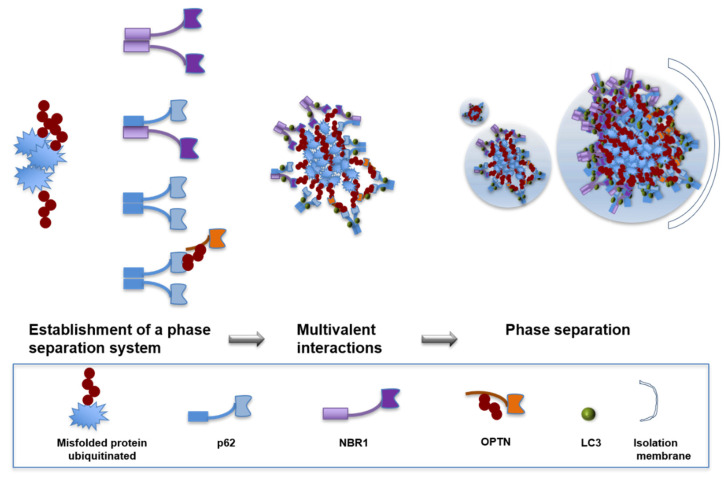
Autophagic cargo segregation through polyubiquitin chain-induced autophagy-receptor phase separation. p62 and NBR1 proteins form oligomers through the PB1 domain (mandarin blue and lavender rectangles) and bind to ubiquitin through the UBA domain (light blue part and deep violet parts). p62 can also bind to the polyubiquitin chains of autophagy receptor OPTN to form autophagy receptor complexes; thus, OPTN can form oligomers through the PB1 domain of P62 and bind to ubiquitin through the UBA domain (orange part). Both domains facilitate multivalent interactions. When protein concentrations reach a threshold, liquid–liquid phase separation occurs to form p62 bodies. Other client proteins, such as LC3, are also recruited to p62 bodies, which are then degraded by autophagy.

Considering the very strong correlation of protein aggregates with human neurodegenerative diseases, it is vital to answer the question of when and how the selective degradation of aggregates is determined. The ubiquitination of substrate proteins is a universal signal to involve the two branches of cellular protein quality control. Since protein aggregates cannot be handled by proteasome degradation, oligomeric autophagy receptors with lower affinity for ubiquitin gain the upper hand through bundling, which ensures that the toxic aggregates are eventually cleared from the cell through the appropriate degradation branch (Figure 5).

In the future, the ubiquitination of different substrates needs to be further clarified. Since proteasome and autophagy pathways share at least partially the same catalytic enzymes, it is not clear whether additional ubiquitination continues after soluble or slightly misfolded proteins accumulate into aggregates. Another noteworthy point may be to determine the sites for these two branches of degradation pathways. As the proteasome is mainly localized in the cell nucleus and autophagy only functions in the cytosol, when and how is the cargo degradation pathway determined? DNA damage stimulates autophagy, and p62 promotes the degradation of nuclear factors for DNA damage response. An additional thorough investigation of the potential link between nuclear events such as the DNA damage response and cytosolic autophagy may expand our insight into cell homeostasis control.

## Data Availability

Not applicable.

## References

[1] Hartl F.U., Hayer-Hartl M. (2009). Converging concepts of protein folding in vitro and in vivo. Nat. Struct. Mol. Biol..

[2] Knowles T.P., Vendruscolo M., Dobson C.M. (2014). The amyloid state and its association with protein misfolding diseases. Nat. Rev. Mol. Cell Biol..

[3] Hipp M.S., Park S.H., Hartl F.U. (2014). Proteostasis impairment in protein-misfolding and -aggregation diseases. Trends Cell Biol..

[4] Feng Y., He D., Yao Z., Klionsky D.J. (2014). The machinery of macroautophagy. Cell Res..

[5] Klionsky D.J., Schulman B.A. (2014). Dynamic regulation of macroautophagy by distinctive ubiquitin-like proteins. Nat. Struct. Mol. Biol..

[6] Khaminets A., Behl C., Dikic I. (2016). Ubiquitin-Dependent And Independent Signals In Selective Autophagy. Trends Cell Biol..

[7] Kirkin V., McEwan D.G., Novak I., Dikic I. (2009). A role for ubiquitin in selective autophagy. Mol. Cell.

[8] Lippai M., Low P. (2014). The role of the selective adaptor p62 and ubiquitin-like proteins in autophagy. Biomed. Res. Int..

[9] Kraft C., Peter M., Hofmann K. (2010). Selective autophagy: Ubiquitin-mediated recognition and beyond. Nat. Cell Biol..

[10] Pankiv S., Clausen T.H., Lamark T., Brech A., Bruun J.A., Outzen H., Overvatn A., Bjorkoy G., Johansen T. (2007). p62/SQSTM1 binds directly to Atg8/LC3 to facilitate degradation of ubiquitinated protein aggregates by autophagy. J. Biol. Chem..

[11] Kirkin V., Lamark T., Sou Y.S., Bjorkoy G., Nunn J.L., Bruun J.A., Shvets E., McEwan D.G., Clausen T.H., Wild P. (2009). A role for NBR1 in autophagosomal degradation of ubiquitinated substrates. Mol. Cell.

[12] Lu K., Psakhye I., Jentsch S. (2014). Autophagic clearance of polyQ proteins mediated by ubiquitin-Atg8 adaptors of the conserved CUET protein family. Cell.

[13] Chen X., Randles L., Shi K., Tarasov S.G., Aihara H., Walters K.J. (2016). Structures of Rpn1 T1:Rad23 and hRpn13:hPLIC2 Reveal Distinct Binding Mechanisms between Substrate Receptors and Shuttle Factors of the Proteasome. Structure.

[14] Liang R.Y., Chen L., Ko B.T., Shen Y.H., Li Y.T., Chen B.R., Lin K.T., Madura K., Chuang S.M. (2014). Rad23 interaction with the proteasome is regulated by phosphorylation of its ubiquitin-like (UbL) domain. J. Mol. Biol..

[15] Lambertson D., Chen L., Madura K. (2003). Investigating the importance of proteasome-interaction for Rad23 function. Curr. Genet..

[16] Davies S.E., Hallett P.J., Moens T., Smith G., Mangano E., Kim H.T., Goldberg A.L., Liu J.L., Isacson O., Tofaris G.K. (2014). Enhanced ubiquitin-dependent degradation by Nedd4 protects against alpha-synuclein accumulation and toxicity in animal models of Parkinson’s disease. Neurobiol. Dis..

[17] Lee J., Kim H.R., Quinley C., Kim J., Gonzalez-Navajas J., Xavier R., Raz E. (2012). Autophagy suppresses interleukin-1beta (IL-1beta) signaling by activation of p62 degradation via lysosomal and proteasomal pathways. J. Biol. Chem..

[18] Stolz A., Ernst A., Dikic I. (2014). Cargo recognition and trafficking in selective autophagy. Nat. Cell Biol..

[19] Tan J.M., Wong E.S., Kirkpatrick D.S., Pletnikova O., Ko H.S., Tay S.P., Ho M.W., Troncoso J., Gygi S.P., Lee M.K. (2008). Lysine 63-linked ubiquitination promotes the formation and autophagic clearance of protein inclusions associated with neurodegenerative diseases. Hum. Mol. Genet..

[20] Lim G.G., Chew K.C., Ng X.H., Henry-Basil A., Sim R.W., Tan J.M., Chai C., Lim K.L. (2013). Proteasome inhibition promotes Parkin-Ubc13 interaction and lysine 63-linked ubiquitination. PLoS ONE.

[21] Riley B.E., Kaiser S.E., Shaler T.A., Ng A.C., Hara T., Hipp M.S., Lage K., Xavier R.J., Ryu K.Y., Taguchi K. (2010). Ubiquitin accumulation in autophagy-deficient mice is dependent on the Nrf2-mediated stress response pathway: A potential role for protein aggregation in autophagic substrate selection. J. Cell Biol..

[22] Ben Yehuda A., Risheq M., Novoplansky O., Bersuker K., Kopito R.R., Goldberg M., Brandeis M. (2017). Ubiquitin Accumulation on Disease Associated Protein Aggregates is Correlated with Nuclear Ubiquitin Depletion, Histone De-Ubiquitination and Impaired DNA Damage Response. PLoS ONE.

[23] Liu Z., Chen P., Gao H., Gu Y., Yang J., Peng H., Xu X., Wang H., Yang M., Liu X. (2014). Ubiquitylation of autophagy receptor Optineurin by HACE1 activates selective autophagy for tumor suppression. Cancer Cell.

[24] Ohsumi Y. (2014). Historical landmarks of autophagy research. Cell Res..

[25] Klionsky D.J. (2008). Autophagy revisited: A conversation with Christian de Duve. Autophagy.

[26] Filimonenko M., Isakson P., Finley K.D., Anderson M., Jeong H., Melia T.J., Bartlett B.J., Myers K.M., Birkeland H.C., Lamark T. (2010). The selective macroautophagic degradation of aggregated proteins requires the PI3P-binding protein Alfy. Mol. Cell.

[27] Bolender R.P., Weibel E.R. (1973). A morphometric study of the removal of phenobarbital-induced membranes from hepatocytes after cessation of threatment. J. Cell Biol..

[28] Veenhuis M., Douma A., Harder W., Osumi M. (1983). Degradation and turnover of peroxisomes in the yeast Hansenula polymorpha induced by selective inactivation of peroxisomal enzymes. Arch. Microbiol..

[29] McElligott M.A., Dice J.F. (1985). Degradation of microinjected ribonuclease A and ribonuclease S-protein by lysosomal pathways. Prog. Clin. Biol. Res..

[30] Mortimore G.E., Poso A.R., Kadowaki M., Wert J.J. (1987). Multiphasic control of hepatic protein degradation by regulatory amino acids. General features and hormonal modulation. J. Biol. Chem..

[31] Lemasters J.J. (2014). Variants of mitochondrial autophagy: Types 1 and 2 mitophagy and micromitophagy (Type 3). Redox Biol..

[32] Nakagawa I., Amano A., Mizushima N., Yamamoto A., Yamaguchi H., Kamimoto T., Nara A., Funao J., Nakata M., Tsuda K. (2004). Autophagy defends cells against invading group A Streptococcus. Science.

[33] Singh S.B., Davis A.S., Taylor G.A., Deretic V. (2006). Human IRGM induces autophagy to eliminate intracellular mycobacteria. Science.

[34] Suzuki K., Kondo C., Morimoto M., Ohsumi Y. (2010). Selective transport of alpha-mannosidase by autophagic pathways: Identification of a novel receptor, Atg34p. J. Biol. Chem..

[35] Kirkin V. (2020). History of the Selective Autophagy Research: How Did It Begin and Where Does It Stand Today?. J. Mol. Biol..

[36] Vadlamudi R.K., Shin J. (1998). Genomic structure and promoter analysis of the p62 gene encoding a non-proteasomal multiubiquitin chain binding protein. FEBS Lett..

[37] Fujioka Y., Suzuki S.W., Yamamoto H., Kondo-Kakuta C., Kimura Y., Hirano H., Akada R., Inagaki F., Ohsumi Y., Noda N.N. (2014). Structural basis of starvation-induced assembly of the autophagy initiation complex. Nat. Struct. Mol. Biol..

[38] Deretic V. (2009). Links between autophagy, innate immunity, inflammation and Crohn’s disease. Dig. Dis..

[39] Brest P., Corcelle E.A., Cesaro A., Chargui A., Belaid A., Klionsky D.J., Vouret-Craviari V., Hebuterne X., Hofman P., Mograbi B. (2010). Autophagy and Crohn′s disease: At the crossroads of infection, inflammation, immunity, and cancer. Curr. Mol. Med..

[40] Cappelletti C., Galbardi B., Kapetis D., Vattemi G., Guglielmi V., Tonin P., Salerno F., Morandi L., Tomelleri G., Mantegazza R. (2014). Autophagy, inflammation and innate immunity in inflammatory myopathies. PLoS ONE.

[41] Zhong Z., Sanchez-Lopez E., Karin M. (2016). Autophagy, Inflammation, and Immunity: A Troika Governing Cancer and Its Treatment. Cell.

[42] Nakatogawa H., Suzuki K., Kamada Y., Ohsumi Y. (2009). Dynamics and diversity in autophagy mechanisms: Lessons from yeast. Nat. Rev. Mol. Cell Biol..

[43] Suzuki H., Osawa T., Fujioka Y., Noda N.N. (2017). Structural biology of the core autophagy machinery. Curr. Opin. Struct. Biol..

[44] Wen X., Klionsky D.J. (2016). An overview of macroautophagy in yeast. J. Mol. Biol..

[45] Matoba K., Kotani T., Tsutsumi A., Tsuji T., Mori T., Noshiro D., Sugita Y., Nomura N., Iwata S., Ohsumi Y. (2020). Atg9 is a lipid scramblase that mediates autophagosomal membrane expansion. Nat. Struct. Mol. Biol..

[46] Yin Z., Pascual C., Klionsky D.J. (2016). Autophagy: Machinery and regulation. Microb. Cell.

[47] Li W., Yang Q., Mao Z. (2011). Chaperone-mediated autophagy: Machinery, regulation and biological consequences. Cell Mol. Life Sci..

[48] Yang Z., Klionsky D.J. (2010). Mammalian autophagy: Core molecular machinery and signaling regulation. Curr. Opin. Cell Biol..

[49] Periyasamy-Thandavan S., Jiang M., Schoenlein P., Dong Z. (2009). Autophagy: Molecular machinery, regulation, and implications for renal pathophysiology. Am. J. Physiol. Renal. Physiol..

[50] Mizushima N., Noda T., Yoshimori T., Tanaka Y., Ishii T., George M.D., Klionsky D.J., Ohsumi M., Ohsumi Y. (1998). A protein conjugation system essential for autophagy. Nature.

[51] Martens S., Fracchiolla D. (2020). Activation and targeting of ATG8 protein lipidation. Cell Discov..

[52] Collier J.J., Guissart C., Oláhová M., Sasorith S., Piron-Prunier F., Suomi F., Zhang D., Martinez-Lopez N., Leboucq N., Bahr A. (2021). Developmental Consequences of Defective ATG7-Mediated Autophagy in Humans. N. Engl. J. Med..

[53] Nguyen T.N., Padman B.S., Usher J., Oorschot V., Ramm G., Lazarou M. (2016). Atg8 family LC3/GABARAP proteins are crucial for autophagosome-lysosome fusion but not autophagosome formation during PINK1/Parkin mitophagy and starvation. J. Cell Biol..

[54] Ohnstad A.E., Delgado J.M., North B.J., Nasa I., Kettenbach A.N., Schultz S.W., Shoemaker C.J. (2020). Receptor-mediated clustering of FIP200 bypasses the role of LC3 lipidation in autophagy. EMBO J..

[55] Lefebvre C., Legouis R., Culetto E. (2018). ESCRT and autophagies: Endosomal functions and beyond. Semin. Cell Dev. Biol..

[56] Zhou F., Wu Z., Zhao M., Murtazina R., Cai J., Zhang A., Li R., Sun D., Li W., Zhao L. (2019). Rab5-dependent autophagosome closure by ESCRT. J. Cell Biol..

[57] Zhou F., Wu Z., Zhao M., Segev N., Liang Y. (2019). Autophagosome closure by ESCRT: Vps21/RAB5-regulated ESCRT recruitment via an Atg17-Snf7 interaction. Autophagy.

[58] Zhen Y., Radulovic M., Vietri M., Stenmark H. (2021). Sealing holes in cellular membranes. EMBO J..

[59] Takahashi Y., He H., Tang Z., Hattori T., Liu Y., Young M.M., Serfass J.M., Chen L., Gebru M., Chen C. (2018). An autophagy assay reveals the ESCRT-III component CHMP2A as a regulator of phagophore closure. Nat. Commun..

[60] Zhen Y., Spangenberg H., Munson M.J., Brech A., Schink K.O., Tan K.W., Sørensen V., Wenzel E.M., Radulovic M., Engedal N. (2020). ESCRT-mediated phagophore sealing during mitophagy. Autophagy.

[61] Fatyol K., Grummt I. (2008). Proteasomal ATPases are associated with rDNA: The ubiquitin proteasome system plays a direct role in RNA polymerase I transcription. Biochim. Biophys. Acta.

[62] Domingues A.F., Arduino D.M., Esteves A.R., Swerdlow R.H., Oliveira C.R., Cardoso S.M. (2008). Mitochondria and ubiquitin-proteasomal system interplay: Relevance to Parkinson′s disease. Free Radic. Biol. Med..

[63] Taylor J.M., Song Y.J., Huang Y., Farrer M.J., Delatycki M.B., Halliday G.M., Lockhart P.J. (2007). Parkin Co-Regulated Gene (PACRG) is regulated by the ubiquitin-proteasomal system and is present in the pathological features of Parkinsonian diseases. NeuroBiol. Dis..

[64] Pfirrmann T., Jandt E., Ranft S., Lokapally A., Neuhaus H., Perron M., Hollemann T. (2016). Hedgehog-dependent E3-ligase Midline1 regulates ubiquitin-mediated proteasomal degradation of Pax6 during visual system development. Proc. Natl. Acad. Sci. USA.

[65] Gadhave K., Bolshette N., Ahire A., Pardeshi R., Thakur K., Trandafir C., Istrate A., Ahmed S., Lahkar M., Muresanu D.F. (2016). The ubiquitin proteasomal system: A potential target for the management of Alzheimer′s disease. J. Cell Mol. Med..

[66] Chakraborty J., Rajamma U., Jana N., Mohanakumar K.P. (2015). Quercetin improves the activity of the ubiquitin-proteasomal system in 150Q mutated huntingtin-expressing cells but exerts detrimental effects on neuronal survivability. J. Neurosci. Res..

[67] Kastle M., Grune T. (2011). Protein oxidative modification in the aging organism and the role of the ubiquitin proteasomal system. Curr. Pharm. Des..

[68] Cao B., Mao X. (2011). The ubiquitin-proteasomal system is critical for multiple myeloma: Implications in drug discovery. Am. J. Blood Res..

[69] Ryabovol V.V., Minibayeva F.V. (2016). Molecular Mechanisms of Autophagy in Plants: Role of ATG8 Proteins in Formation and Functioning of Autophagosomes. Biochemistry.

[70] Kellner R., De la Concepcion J.C., Maqbool A., Kamoun S., Dagdas Y.F. (2017). ATG8 Expansion: A Driver of Selective Autophagy Diversification?. Trends Plant. Sci..

[71] Fracchiolla D., Zens B., Martens S. (2017). In Vitro Reconstitution of Atg8 Conjugation and Deconjugation. Methods Enzymol..

[72] Abreu S., Kriegenburg F., Gomez-Sanchez R., Mari M., Sanchez-Wandelmer J., Skytte Rasmussen M., Soares Guimaraes R., Zens B., Schuschnig M., Hardenberg R. (2017). Conserved Atg8 recognition sites mediate Atg4 association with autophagosomal membranes and Atg8 deconjugation. EMBO Rep..

[73] Voss C., Ehrenman K., Mlambo G., Mishra S., Kumar K.A., Sacci J.B., Sinnis P., Coppens I. (2016). Overexpression of Plasmodium berghei ATG8 by Liver Forms Leads to Cumulative Defects in Organelle Dynamics and to Generation of Noninfectious Merozoites. MBio.

[74] Bjorkoy G., Lamark T., Brech A., Outzen H., Perander M., Overvatn A., Stenmark H., Johansen T. (2005). p62/SQSTM1 forms protein aggregates degraded by autophagy and has a protective effect on huntingtin-induced cell death. J. Cell Biol..

[75] Komatsu M., Waguri S., Koike M., Sou Y.S., Ueno T., Hara T., Mizushima N., Iwata J., Ezaki J., Murata S. (2007). Homeostatic levels of p62 control cytoplasmic inclusion body formation in autophagy-deficient mice. Cell.

[76] Gal J., Strom A.L., Kilty R., Zhang F., Zhu H. (2007). p62 accumulates and enhances aggregate formation in model systems of familial amyotrophic lateral sclerosis. J. Biol. Chem..

[77] Slowicka K., Vereecke L., Mc Guire C., Sze M., Maelfait J., Kolpe A., Saelens X., Beyaert R., van Loo G. (2016). Optineurin deficiency in mice is associated with increased sensitivity to Salmonella but does not affect proinflammatory NF-kappaB signaling. Eur. J. Immunol..

[78] Chew T.S., O’Shea N.R., Sewell G.W., Oehlers S.H., Mulvey C.M., Crosier P.S., Godovac-Zimmermann J., Bloom S.L., Smith A.M., Segal A.W. (2015). Optineurin deficiency in mice contributes to impaired cytokine secretion and neutrophil recruitment in bacteria-driven colitis. Dis. Model. Mech..

[79] Kim B.W., Hong S.B., Kim J.H., Kwon D.H., Song H.K. (2013). Structural basis for recognition of autophagic receptor NDP52 by the sugar receptor galectin-8. Nat. Commun..

[80] Lu K., Psakhye I., Jentsch S. (2014). A new class of ubiquitin-Atg8 receptors involved in selective autophagy and polyQ protein clearance. Autophagy.

[81] Lu K., den Brave F., Jentsch S. (2017). Receptor oligomerization guides pathway choice between proteasomal and autophagic degradation. Nat. Cell Biol..

[82] Sarraf S.A., Shah H.V., Kanfer G., Pickrell A.M., Holtzclaw L.A., Ward M.E., Youle R.J. (2020). Loss of TAX1BP1-Directed Autophagy Results in Protein Aggregate Accumulation in the Brain. Mol. Cell.

[83] Liu W.J., Ye L., Huang W.F., Guo L.J., Xu Z.G., Wu H.L., Yang C., Liu H.F. (2016). p62 links the autophagy pathway and the ubiqutin-proteasome system upon ubiquitinated protein degradation. Cell Mol. Biol. Lett..

[84] Hewitt G., Carroll B., Sarallah R., Correia-Melo C., Ogrodnik M., Nelson G., Otten E.G., Manni D., Antrobus R., Morgan B.A. (2016). SQSTM1/p62 mediates crosstalk between autophagy and the UPS in DNA repair. Autophagy.

[85] Deng Z., Purtell K., Lachance V., Wold M.S., Chen S., Yue Z. (2017). Autophagy Receptors and Neurodegenerative Diseases. Trends Cell Biol..

[86] Matsumoto G., Wada K., Okuno M., Kurosawa M., Nukina N. (2011). Serine 403 phosphorylation of p62/SQSTM1 regulates selective autophagic clearance of ubiquitinated proteins. Mol. Cell.

[87] Richter B., Sliter D.A., Herhaus L., Stolz A., Wang C., Beli P., Zaffagnini G., Wild P., Martens S., Wagner S.A. (2016). Phosphorylation of OPTN by TBK1 enhances its binding to Ub chains and promotes selective autophagy of damaged mitochondria. Proc. Natl. Acad. Sci. USA.

[88] Wild P., Farhan H., McEwan D.G., Wagner S., Rogov V.V., Brady N.R., Richter B., Korac J., Waidmann O., Choudhary C. (2011). Phosphorylation of the autophagy receptor optineurin restricts Salmonella growth. Science.

[89] O’Loughlin T., Kruppa A.J., Ribeiro A.L.R., Edgar J.R., Ghannam A., Smith A.M., Buss F. (2020). OPTN recruitment to a Golgi-proximal compartment regulates immune signalling and cytokine secretion. J. Cell Sci..

[90] Moore A.S., Holzbaur E.L. (2016). Dynamic recruitment and activation of ALS-associated TBK1 with its target optineurin are required for efficient mitophagy. Proc. Natl. Acad. Sci. USA.

[91] Tumbarello D.A., Manna P.T., Allen M., Bycroft M., Arden S.D., Kendrick-Jones J., Buss F. (2015). The Autophagy Receptor TAX1BP1 and the Molecular Motor Myosin VI Are Required for Clearance of Salmonella Typhimurium by Autophagy. PLoS Pathog..

[92] Verstrepen L., Verhelst K., Carpentier I., Beyaert R. (2011). TAX1BP1, a ubiquitin-binding adaptor protein in innate immunity and beyond. Trends Biochem. Sci..

[93] Gurung R., Tan A., Ooms L.M., McGrath M.J., Huysmans R.D., Munday A.D., Prescott M., Whisstock J.C., Mitchell C.A. (2003). Identification of a novel domain in two mammalian inositol-polyphosphate 5-phosphatases that mediates membrane ruffle localization. The inositol 5-phosphatase skip localizes to the endoplasmic reticulum and translocates to membrane ruffles following epidermal growth factor stimulation. J. Biol. Chem..

[94] Yang Y., Wang G., Huang X., Du Z. (2014). Expression, purification and crystallization of the SKICH domain of human TAX1BP1. Acta Crystallogr. F Struct. Biol. Commun..

[95] Iha H., Peloponese J.M., Verstrepen L., Zapart G., Ikeda F., Smith C.D., Starost M.F., Yedavalli V., Heyninck K., Dikic I. (2008). Inflammatory cardiac valvulitis in TAX1BP1-deficient mice through selective NF-kappaB activation. EMBO J..

[96] Ceregido M.A., Spinola Amilibia M., Buts L., Rivera-Torres J., Garcia-Pino A., Bravo J., van Nuland N.A. (2014). The structure of TAX1BP1 UBZ1+2 provides insight into target specificity and adaptability. J. Mol. Biol..

[97] De Valck D., Jin D.Y., Heyninck K., Van de Craen M., Contreras R., Fiers W., Jeang K.T., Beyaert R. (1999). The zinc finger protein A20 interacts with a novel anti-apoptotic protein which is cleaved by specific caspases. Oncogene.

[98] Toruń A., Szymańska E., Castanon I., Wolińska-Nizioł L., Bartosik A., Jastrzębski K., Miętkowska M., Gonzalez-Gaitan M., Miaczynska M. (2015). Endocytic Adaptor Protein Tollip Inhibits Canonical Wnt Signaling. PLoS ONE.

[99] Burns K., Clatworthy J., Martin L., Martinon F., Plumpton C., Maschera B., Lewis A., Ray K., Tschopp J., Volpe F. (2000). Tollip, a new component of the IL-1RI pathway, links IRAK to the IL-1 receptor. Nat. Cell Biol..

[100] Prag G., Misra S., Jones E.A., Ghirlando R., Davies B.A., Horazdovsky B.F., Hurley J.H. (2003). Mechanism of ubiquitin recognition by the CUE domain of Vps9p. Cell.

[101] Carroll B., Otten E.G., Manni D., Stefanatos R., Menzies F.M., Smith G.R., Jurk D., Kenneth N., Wilkinson S., Passos J.F. (2018). Oxidation of SQSTM1/p62 mediates the link between redox state and protein homeostasis. Nat. Commun..

[102] Svenning S., Lamark T., Krause K., Johansen T. (2011). Plant NBR1 is a selective autophagy substrate and a functional hybrid of the mammalian autophagic adapters NBR1 and p62/SQSTM1. Autophagy.

[103] Hirano S., Uemura T., Annoh H., Fujita N., Waguri S., Itoh T., Fukuda M. (2016). Differing susceptibility to autophagic degradation of two LC3-binding proteins: SQSTM1/p62 and TBC1D25/OATL1. Autophagy.

[104] Yamasaki A., Watanabe Y., Adachi W., Suzuki K., Matoba K., Kirisako H., Kumeta H., Nakatogawa H., Ohsumi Y., Inagaki F. (2016). Structural Basis for Receptor-Mediated Selective Autophagy of Aminopeptidase I Aggregates. Cell Rep..

[105] Bertipaglia C., Schneider S., Jakobi A.J., Tarafder A.K., Bykov Y.S., Picco A., Kukulski W., Kosinski J., Hagen W.J., Ravichandran A.C. (2016). Higher-order assemblies of oligomeric cargo receptor complexes form the membrane scaffold of the Cvt vesicle. EMBO Rep..

[106] Yamasaki A., Noda N.N. (2017). Structural Biology of the Cvt Pathway. J. Mol. Biol..

[107] Sun D., Wu R., Li P., Yu L. (2020). Phase Separation in Regulation of Aggrephagy. J. Mol. Biol..

[108] Banani S.F., Lee H.O., Hyman A.A., Rosen M.K. (2017). Biomolecular condensates: Organizers of cellular biochemistry. Nat. Rev. Mol. Cell Biol..

[109] Li P., Banjade S., Cheng H.C., Kim S., Chen B., Guo L., Llaguno M., Hollingsworth J.V., King D.S., Banani S.F. (2012). Phase transitions in the assembly of multivalent signalling proteins. Nature.

[110] Wilson M.I., Gill D.J., Perisic O., Quinn M.T., Williams R.L. (2003). PB1 domain-mediated heterodimerization in NADPH oxidase and signaling complexes of atypical protein kinase C with Par6 and p62. Mol. Cell.

[111] Sun D., Wu R., Zheng J., Li P., Yu L. (2018). Polyubiquitin chain-induced p62 phase separation drives autophagic cargo segregation. Cell Res..

[112] Altmeyer M., Neelsen K.J., Teloni F., Pozdnyakova I., Pellegrino S., Grøfte M., Rask M.B.D., Streicher W., Jungmichel S., Nielsen M.L. (2015). Liquid demixing of intrinsically disordered proteins is seeded by poly(ADP-ribose). Nat. Commun..

[113] Agudo-Canalejo J., Schultz S.W., Chino H., Migliano S.M., Saito C., Koyama-Honda I., Stenmark H., Brech A., May A.I., Mizushima N. (2021). Wetting regulates autophagy of phase-separated compartments and the cytosol. Nature.

[114] Kirkin V., Lamark T., Johansen T., Dikic I. (2009). NBR1 cooperates with p62 in selective autophagy of ubiquitinated targets. Autophagy.

[115] Itakura E., Mizushima N. (2011). p62 Targeting to the autophagosome formation site requires self-oligomerization but not LC3 binding. J. Cell Biol..

[116] Hjerpe R., Bett J.S., Keuss M.J., Solovyova A., McWilliams T.G., Johnson C., Sahu I., Varghese J., Wood N., Wightman M. (2016). UBQLN2 Mediates Autophagy-Independent Protein Aggregate Clearance by the Proteasome. Cell.

[117] Deng H.X., Chen W., Hong S.T., Boycott K.M., Gorrie G.H., Siddique N., Yang Y., Fecto F., Shi Y., Zhai H. (2011). Mutations in UBQLN2 cause dominant X-linked juvenile and adult-onset ALS and ALS/dementia. Nature.

[118] Zhang K.Y., Yang S., Warraich S.T., Blair I.P. (2014). Ubiquilin 2: A component of the ubiquitin-proteasome system with an emerging role in neurodegeneration. Int. J. Biochem. Cell Biol..

